# Strain-Level Differences of *Bifidobacterium breve* in the Gut Microbiome between Infants with and without Atopic Dermatitis: Insights from Genome Analysis and Immune Assays

**DOI:** 10.4014/jmb.2509.09032

**Published:** 2025-11-26

**Authors:** Imchang Lee, Seong Hee Kim, Min-Jung Lee, Ara Oh, Yun Kyung Lee, Kwang Jun Lee, Bong-Soo Kim

**Affiliations:** 1 Division of Infectious Diseases, Department of Internal Medicine, Hallym University Chuncheon Sacred Heart Hospital, Hallym Univeristy College of Medicine, Chuncheon 24252, Republic of Korea; 2 Department of Life Science, Hallym University, Chuncheon 24252, Republic of Korea; 3 Department of Nutritional Science and Food Management, Ewha Womans University, Seoul 03760, Republic of Korea; 4 Department of Integrated Biomedical Science, Soonchunhyang Institute of Medi-Bioscience, Soonchunhyang University, Cheonan 31151, Republic of Korea; 5 Division of Zoonotic and Vector Brone Disease Research, National Institute of Health, Korea Disease Control and Prevention Agency, Cheongju 28160, Republic of Korea

**Keywords:** *Bifidobacterium breve*, strain difference, probiotics, genome, immune assay

## Abstract

*Bifidobacterium breve* is recognized as a probiotic with immune-enhancing properties. However, our previous studies revealed that this species is present at a higher relative abundance in the gut microbiome of infants with atopic dermatitis (AD). The potential functions of *B. breve* in the gut microbiome may vary at the strain level between infants with and without AD (non-AD). In this study, *B. breve* strains were isolated from the feces of infants with and without AD and analyzed whole genome sequencing and immune assay to identify strain level differences between AD and non-AD groups. Three *B. breve* strains were isolated from the feces of infants with AD (MHL_0001), in remission (MHL_0043), and non-AD infants (MHL_0062). The genomes of these isolates were compared to available complete genomes of *B. breve* strains. While the three isolates exhibited high overall genome similarity, differences in the sequence homology of immune related genes were observed between the AD strain (MHL_0001) and non-AD strain (MHL_0062). Immune assays further revealed marked differences in the anti-inflammatory effects between MHL_0001 and MHL_0062. These findings suggest that probiotic bacteria such as *B. breve* may adapt within the gut in response to host immune and physiological conditions. Moreover, the presence of *B. breve* in the gut microbiome does not necessarily guarantee beneficial effects for the host. Therefore, strain-level analysis is crucial to accurately determine the functional roles and impact of probiotic bacteria.

## Introduction

Atopic dermatitis (AD) is a chronic inflammatory skin disorder characterized by intense itching, erythema, and xerosis. Typically beginning in early childhood, AD can persist into adulthood, profoundly affecting the quality of life [1]. An imbalance in the immune response, particularly Th_2_-skewed inflammation with elevated levels of cytokines such as IL-4, IL-5, and IL-13, plays a crucial role in the pathophysiology of AD [2]. The gut microbiome in infants is known to influence immune system development and modulate Th_2_ inflammation through the induction of regulatory T (T_reg_) cells [3-5]. Several studies have demonstrated that alterations in the gut microbiome affect the severity and progression of AD by disrupting short-chain fatty acid (SCFA) production and immune homeostasis [6-9].

*Bifidobacterium* is a dominant genus in the gut microbiota of infants, with its abundances decreasing in weaned infants and adults [10, 11]. *Bifidobacterium* spp. is among the first microbial colonizers in the gut of breastfed infants, playing a pivotal role in shaping the gut ecosystem with potential long-term health consequences [4]. Studies have shown that atopic infants exhibit a higher *Clostridia* to *Bifidobacterium* ratio compared to non-atopic infants [12]. Accordingly, several studies have highlighted the significant role of *Bifidobacterium* in the gut microbiome, noting differences in its relative abundance between infants with and without AD [6, 13-15]. However, other studies have reported no significant differences in the relative abundances of *Bifidobacterium* between groups [7, 9, 16-19]. Moreover, different *Bifidobacterium* species may have distinct roles in allergic diseases. For example, *Bifidobacterium breve*, an infant-type bifidobacteria, has been linked to a reduced risk of eczema, while lower abundance of this species relative to adult-type species such as *B. pseudocatenulatum* and *B. catenulatum* have been implicated in eczema development [20]. Our previous studies also found that while the relative abundances of *Bifidobacterium* did not significantly differ between infants with and without AD, its abundance was influenced by breastfeeding during early infancy [7, 9].

*B. breve* has attracted significant attention for its potential therapeutic effects, particularly in modulating immune responses and reducing inflammation [21]. The immune benefits of *B. breve* have been demonstrated in infants with allergic disorders [22]. Supplementation with *B. breve* has been shown to increase in T_reg_ cells and decrease pro-inflammatory cytokines, both of which are crucial for managing the chronic inflammation associated with AD [23, 24]. However, despite these benefits, our previous studies revealed a high abundance of *B. breve* in the gut microbiome of infants with AD [7, 9]. These findings suggest that *B. breve* strains may differ between the gut microbiome of infants with and without AD, potentially leading to distinct functional characteristics. Consequently, the immunomodulatory effects of *B. breve* may be diminished or insignificant in infants with AD, despite its considerable prevalence in their gut microbiome.

In this study, we isolated *B. breve* strains from the feces of infants with and without AD to identify the strain-specific differences between the two groups. Genomic differences among the isolated strains were analyzed based on AD status, and the anti-inflammatory effects of each isolate were evaluated using *in vitro* assays.

## Materials and Methods

### Comparison of *Bifidobacterium* in the Gut Microbiome between AD and Non-AD Groups

Whole metagenome sequences of the gut microbiome from 366 infants with (AD) and without AD (non-AD) were obtained from our previous studies [7, 9]. Adapter removal, quality filtering, and human gene contamination trimming were performed as previously described. Species and strain identification were carried out using MetaPhlAn4 v.4.0 [25] and StrainPhlan4 v.4.0 [26], respectively. A phylogenetic distance matrix, based on pairwise nucleotide substitution rate between strains, was used to generate a principal coordinate analysis (PCoA) plot. Group differences were assessed for significance using PERMANOVA (Adonis from the package vegan, with 999 permutation). The significance of differences in relative abundances between AD and non-AD groups was determined using the Wilcoxon-rank sum test and Dunn’s multiple comparison test.

### Isolation of *Bifidobacterium* Strains from the Infant Feces

Collected fecal samples (aged 23-48 months old; 3 non-AD, 3 moderate-severe AD, and 3 AD remission infants) in our previous study [9] were used to isolate *Bifidobacterium* strains. Fecal samples (1 g) were diluted in 1 ml of phosphate-buffered saline, and the diluted samples were plated in triplicate on de Man, Rogosa, and Sharpe (MRS) medium or Tryptic Soy Agar (TSA) medium. Plates were incubated under anaerobic conditions (80% nitrogen, 10% carbon dioxide, and 10% hydrogen) in a Whitley DG250 Anaerobic Workstation (Don Whitley Scientific, UK) at 37°C for 48 h. Colonies were subcultured on the same medium to obtain pure cultures. Bacterial DNA was extracted from pure cultured colonies using the DNeasy UltraClean Microbial Kit (Qiagen, USA) according to the manufacturer's instructions. The 16S rRNA gene was amplified using a C1000 thermal cycler (Bio-Rad, USA) with primers 27F (5'-AGAGTTTGATCCTGGCTCAG-3') and 1492R (5'-GGTTACCTTGTTACGACTT-3'). The resulting amplicons were purified using the QIAquick PCR Purification Kit (Qiagen) and sequenced using an Applied Biosystems ABI3730XL automatic sequencer at Macrogen Corp. (Republic of Korea). Identification of the sequenced 16S rRNA genes was conducted using the EzBioCloud server [27]. *Bifidobacterium* strains were subsequently subcultured in MRS medium under anaerobic conditions for whole genome sequencing. Identification for subcultured strains was conducted again using whole genome sequence. All identified strains were stored as glycerol stock (20% v/v) at -80°C.

### Whole Genome Sequencing

Total genomic DNA was extracted from *B. breve* isolates (MHL0001, MHL0043, and MHL0062) using the Wizard Genomic DNA Purification Kit (Promega, Japan) according to the manufacturer's protocol. Genomic DNA was fragmented using the NEBNext dsDNA Fragmentase (New England Biolabs, USA), and sequencing libraries were prepared with the Accel-NGS 2Plus DNA Library Kit (Swift Biosciences, USA) following the manufacturer's instructions. The prepared libraries were sequenced using the Illumina MiSeq system (300-bp paired end). The sequence reads were assembled using CLC Genomic Workbench v.8.5.1 with reference mapping. The reference genome sequence of *B. breve* JCM 1192 (RefSeq accession num. GCF_001025175.1) was obtained from the NCBI database.

### Comparative Genome Analysis

The 49 complete *B. breve* genome sequences available in the NCBI database (Table S1) were retrieved in nucleotide FASTA format and used to compare genome features of the isolates obtained in this study with previously reported strains. Genome annotation was performed using the NCBI Prokaryotic Genomic Annotation Pipeline (PGAP) and Prokka (default settings) [28]. Clustered Regularly Interspaced Short Palindromic Repeats (CRISPRs) and associated Cas proteins were identified using CRISPRCasFinder v.4.2.20 (default settings) [29]. Putative horizontal gene transfer (HGT) events were detected with Alien Hunter v.1.7. (default settings) [30], and mobile genetic elements were identified using MobileOG-db v.1.6. (default settings) [31]. Prophage regions were predicted using Phigaro v.2.3.0. (default settings) [32], and dsDNA and ssDNA phages were detected using VirSorter v.2.2.4. (default settings) [33]. Antibiotic resistance genes (ARGs) were identified using the Resistance Gene Identifier (RGI) within the Comprehensive Antibiotic Resistance Database (CARD), applying the ‘Perfect’ detection criterion [34].

### Phylogenomics and Evolutionary Analysis

Average nucleotide identity (ANI) values were calculated to re-identify the isolates by comparison with *B. breve* JCM 1192 (type strain) using OrthoANI [35]. Hierarchical clustering was performed based on ANI values using the unweighted pair group method with arithmetic mean (UPGMA) algorithm. For strain-level evolutionary analysis, a single nucleotide pholymorphism (SNP)-based maximum likelihood (ML) tree was constructed using the PhaME tool v.1.0.2. [36], incorporating 49 complete genomes of *B. breve* and the three isolates. *B. longum* subsp. *longum* JCM 1217 was used as an interspecies outgroup, and *B. breve* NRBB09 served as an intraspecies outgroup in the SNP tree.

### Comparative Analysis of Genes Related to Immune Response

To further analyze immunologically relevant genes in the isolated strains, the genome features of five complete genomes clustered with the three isolates in SNP tree, as well as the genome of *B. breve* UCC 2003 (a strain with well-characterized genome functions) [37, 38]. Fifty-one genes with known immune-related functions, as identified in previous studies, were selected for comparison, and concatenated sequences were generated for multiple alignments (Table S2).

A total of 36 genes were selected from the functional annotation of the UCC2003 genome. These included genes involved in exopolysaccharide (EPS) biosynthesis cluster (EPS1 and EPS2), which are associated with immune modulation during host interaction [39]; colonization-related genes involved in pilus formation; the immune complex-inhibiting endo-beta-N-aceytylglucosaminidase gene [40]; and the diacetylchitobiose uptake system permease gene, which facilitates the uptake and metabolism of chitin-derived oligosaccharides, contributing to microbiota alteration and anti-inflammatory effect [41].

Thirteen additional genes were selected from the JCM 1192 genomes, including genes involved in acetate production [42], the immune-boosting high-affinity zinc uptake system binding protein (*znuA*) [43], the inflammation-modulating voltage-gated ClC-type chloride channel (*clcA*) [44], the tress-protective protein/nucleic acid deglycase 2 (*yhbO*), which mitigates inflammation related to glycation-induced cellular damage [45], and the immune response-associated maltose/maltodextrin-binding protein gene (*malX*) [46].

Lastly, two genes were selected from JR01 genome: spermidine synthase (*speE*), which is linked to anti-inflammatory macrophage activity [47], and serine/threonine-protein kinase (*spkl*), which plays a role in inflammation regulation [48].

The presence of these genes in each genome was determined using Usearch v.11.0.667 [49], with criteria of 100% query coverage and greater than 80% sequence identity. If a gene was absent from a genome, a dash ("-") was inserted into the concatenated sequences to maintain consistent alignment lengths across all genomes. The identified genes were concatenated in the 5' to 3' direction to create a unified sequence for each genome, ensuring that the multiple alignment captured the full range of selected genes.

Among the 51 genes, 27 were commonly detected in the genomes of the three isolated strains. The comparison of these genes among isolates was performed based on sequence identity, with the genes in MHL0062 set as reference for the analysis.

### Co-Culture of Ex Vivo Immune Cells with *B. breve*

Nine-week-old C57BL/6 female mice were purchased from ORIENT Bio (Republic of Korea). Mesenteric lymph node (MLN) cells from C57BL/6 mice were cultured in the medium containing each intact *B. breve* strain at a 1:100 cell to bacteria ratio for 24 or 48 h, as previously described [50]. The cells were cultured in RPMI 1640 medium (Corning, USA) supplemented with 10% FBS (Gibco, Germany), 100 U/ml penicillin-streptomycin (Corning), 1 mM sodium pyruvate (Gibco), 1% MEM non-essential amino acid (Corning), 55μM 2-mercaptoethnol (Gibco), and 750μg/ml gentamycin (Sigma-Aldrich, USA).

### Immune Cell Analysis

To analyze cytokine mRNA expression, cells were co-cultured with *B. breve* strains for 24 h and subsequently harvested using TRIZOL reagent (Invitrogen, USA). Total RNA was extracted and reverse-transcribed into cDNA with the ReverTra Ace qPCR RT Master Mix (TOYOBO, Japan) according to the manufacturer’s protocol. Quantitative real-time PCR (qRT-PCR) was performed using the SYBR Green Real-Time PCR Master Mix Kit (Applied Biosystems, USA) with gene-specific primers (Table S3) on a QuantStudio (Applied Biosystems). Relative expression levels were normalized to *β*-actin and expressed relative to cells cultured without bacteria, which were used as the reference (set to 1).

IL-10 concentrations in culture supernatants were measured after 48 h co-culture of mononuclear leukocyte cells with *B. breve* strains. Measurements were performed using a commercially available IL-10 ELISA kit (Invitrogen), following the manufacturer’s instructions.

For flow cytometry, cells co-cultured with *B. breve* strains for 24 h were collected and stained using the LIVE/DEAD Fixable Violet Dead Cell Stain Kit, APC-Cy7-conjugated anti-CD4, and FITC-conjugated anti-Foxp3 antibodies (all from Invitrogen). Surface and intracellular staining were performed according to standard procedures. Samples were acquired on a MACSQuant Analyzer 10 (Miltenvi Biotech, Germany), and data were analyzed using FlowJo software (BD Biosciences, USA).

## Results

### Strain Difference of *B. breve* in the Gut Microbiome between AD and Non-AD Groups

**Fig. 1:**
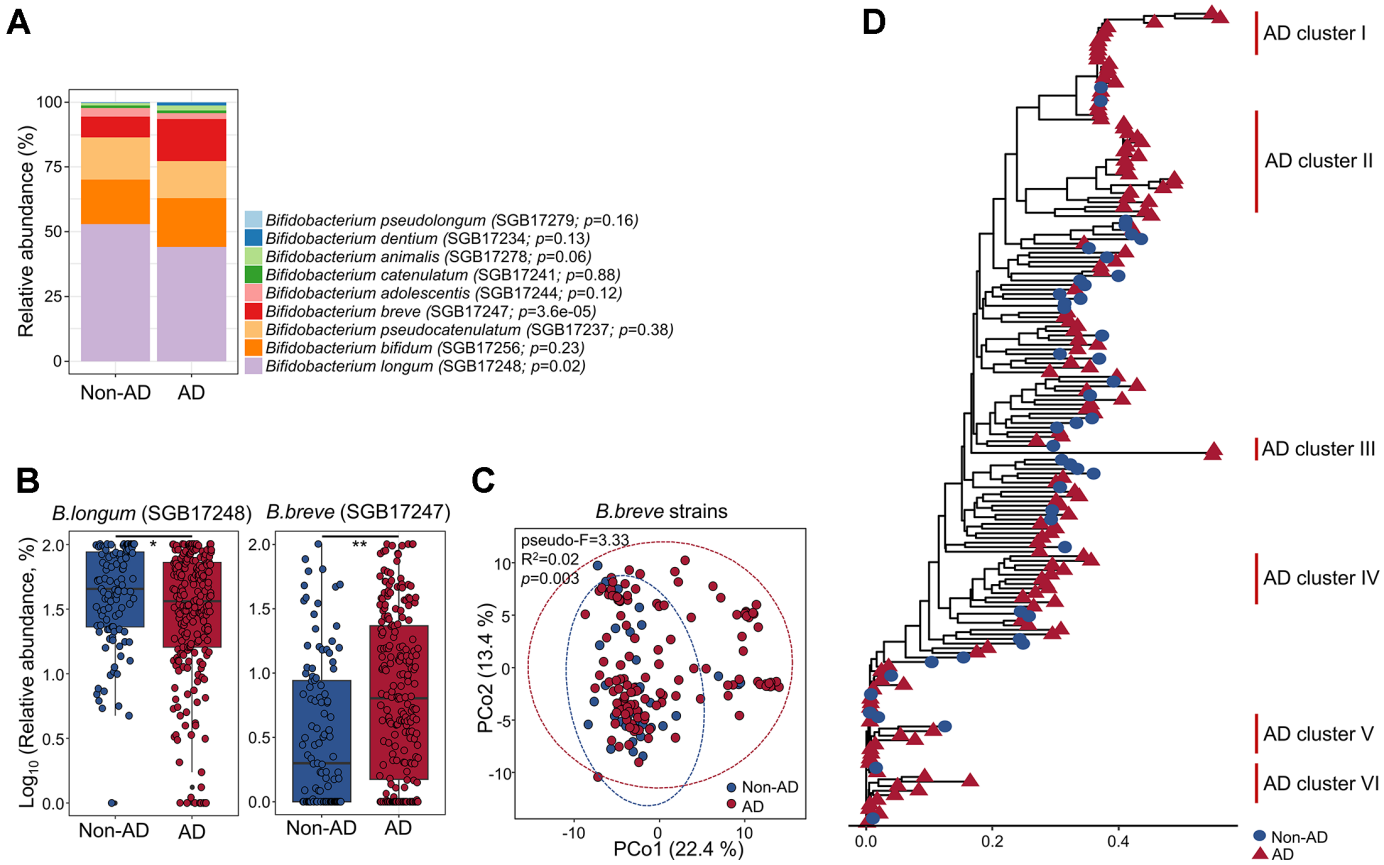
Comparison of *Bifidobacterium* spp. profiles and strain-level variation of *B. breve* in the gut microbiome of infants with and without atopic dermatitis. (**A**) Profiles of *Bifidobacterium* spp. in the gut microbiome were compared between non-AD and AD groups using whole-metagenome data from our previous studies [7, 9]. Taxonomic identification was performed with MetaPhlAn4, and relative abundance was calculated based on taxonomic features. (**B**) Relative abundances of *B. longum* and *B. breve* were compared between the non-AD and AD groups. (**C**) Strain-level variation of *B. breve* between groups were visualized with a PCoA plot based on pairwise nucleotide substitution rates. Strain-level analysis was conducted using StrainPhlan4. (**D**) Phylogenetic tree showing *B. breve* strain differences across samples. Blue circles represent non-AD samples and red triangles represent AD samples. Non-AD, infants without atopic dermatitis; AD, infants with atopic dermatitis; CDS, coding sequences. **p < 0.01, *p < 0.05.

The relative abundance of *Bifidobacterium* in the gut microbiome was compared between AD and non-AD groups using whole metagenome data from our previous study [9]. A total of nine *Bifidobacterium* species were detected in both groups (Fig. 1A; *n* = 366). Among these, *B. breve* and *B. longum* showed significant differences in relative abundance between the two groups (*p* < 0.05). The relative abundance of *B. longum* was higher in the non-AD group than in the AD group, whereas *B. breve* was more abundant in the AD group (Fig. 1B).

The higher abundance of *B. breve* in the AD group compared to the non-AD group suggested potential strain-level differences between the groups. Therefore, we analyzed *B. breve* strain differences using StrainPhlAn v.4.0. Strain-level variation was evident in both the PCoA (pseudo-F = 3.33, R^2^ = 0.02, *p* = 0.003) and the phylogenetic analysis (Fig. 1C and 1D). Monophyletic clades enriched for AD samples in the phylogenetic tree were designated as AD clusters.

### Genome Features of Three *B. breve* Strains Isolated from Infant Feces

To evaluate strain-level differences in *B. breve* between non-AD and AD groups, we isolated *B. breve* strains from fecal samples. Among 80 isolates, one strain from each group was identified as *B. breve*: MHL0001 (severe AD), MHL0043 (remission AD), and MHL0062 (non-AD). Remission refers to the reduction or disappearance of AD symptoms.

**Fig. 2:**
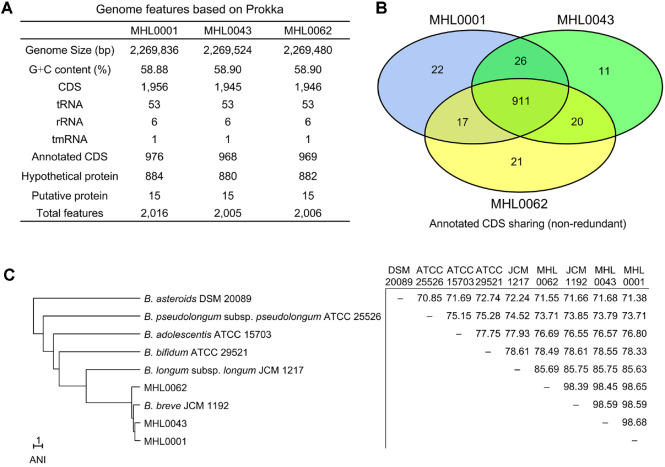
Genome features of isolated *B. breve* strains. (**A**) Genome features of the isolates analyzed using Prokka annotation. (**B**) Venn diagram showing shared annotated coding sequences (CDS) among the three isolates. (**C**) UPGMA tree and distance matrix of the isolates and other *Bifidobacterium* spp., based on average nucleotide identity (ANI). Type species of the *Bifidobacterium* genus were included for ANI calculation. The scale bar denotes a distance equivalent to an ANI value of 1.

The genomes of the three isolates were fully sequenced and successfully assembled into single contigs. Genome features annotated using Prokka are summarized in Fig. 2A. The average genome size was 2,269,613 bp, with an average G+C content of 58.89%. The number of coding sequences (CDS) detected was 1,956 (976 annotated CDS) in MHL0001, 1,945 (968 annotated CDS) in MHL0043, and 1,946 (969 annotated CDS) in MHL0062. Each genome contained the same number of RNA genes: 53 tRNA, 6 rRNA, and 1 tmRNA. A Venn diagram was used to compare the shared and unique annotated CDS among the strains (Fig. 2B). A total of 911 CDS was shared across all strains, while 22 CDS were unique to MHL0001, 11 CDS were unique to MHL0043, and 21 CDS were unique to MHL0062. Unique CDS in each strain and sharing CDS between two strains are summarized in Table S4.

The ANI values of the three isolates were calculated against the type strains of *Bifidobacterium* species. The isolates clustered with *B. breve* JCM 1192 in a UPGMA tree based on ANI value (Fig. 2C). The average ANI value between the three isolates and *B. breve* JCM 1192 was 98.52 ± 0.12, while the ANI values between the isolates themselves averaged 98.59 ± 0.13. The next highest ANI value with *Bifidobacterium* species was detected for *B. longum* subsp. *longum* JCM 1217 (85.69 ± 0.06).

### Comparative Genome Analysis between Available Complete Genomes of *B. breve* and the Isolates

**Fig. 3:**
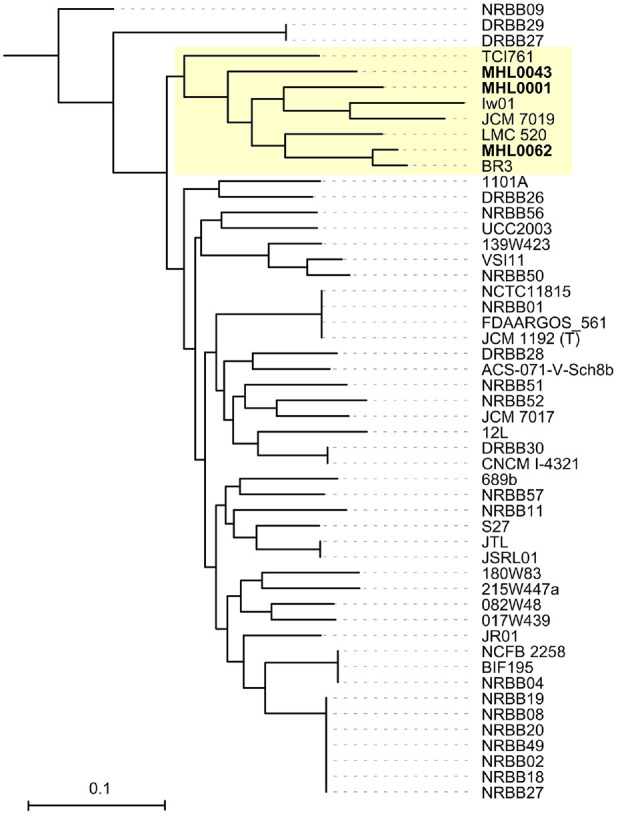
Single nucleotide polymorphism (SNP)-based phylogenetic tree of complete *B. breve* genomes and isolates. Phylogenetic relationships were inferred from SNPs using the Maximum Likelihood (ML) method in Phame. *B. longum* subsp. *longum* JCM 1217 was used as the outgroup. The scale bar indicates substitutions per site. The subclade containing the isolates and previously reported genomes is highlighted in light yellow.

To characterize the genomic features of the *B. breve* strains isolated in this study, a SNP tree was constructed (Fig. 3). The three isolated strains clustered together within a distinct sub-clade, which also included five completed *B. breve* genomes (TCI761, lw01, JCM 7019, LMC 520, and BR3). These genomes, along with the three isolates, were selected for further genome feature analysis. Additionally, strain UCC2003, a widely recognized prototype for *B. breve* species [37, 38, 51], was included in the analysis.

Various genomic components, including CRISPR-Cas system, ARGs, mobile genetic elements, HGT events, and phage-related characteristics, were detected and compared among the strains (Table 1). Genome size ranged from 2.3 to 2.5 Mbp, with an average of 2,009.4 ± 62.4 CDS across the nine strains. CRISPR sequences were detected in all stains, while ARG sequences were absent in all genomes. Mobile elements varied from 95 (lw01) to 151 (BR3), with integration/Excision (IE) and replication/recombination/repair (RRR) elements being the most prevalent. The highest number of predicted HGT events was observed in TCI761 (67), while the lowest was in LMC 520 (39). Prophage and phage-related sequences were detected in all strains.

The genomic features of the isolated strains in this study were highly similar. However, mobile elements, HGT events, and prophage-related sequences were predicted to be higher in MHL0062 compared to the other isolates.

### Comparison of Immune-Related Genes in Genomes of Strains and Isolates within Subclade

**Fig. 4:**
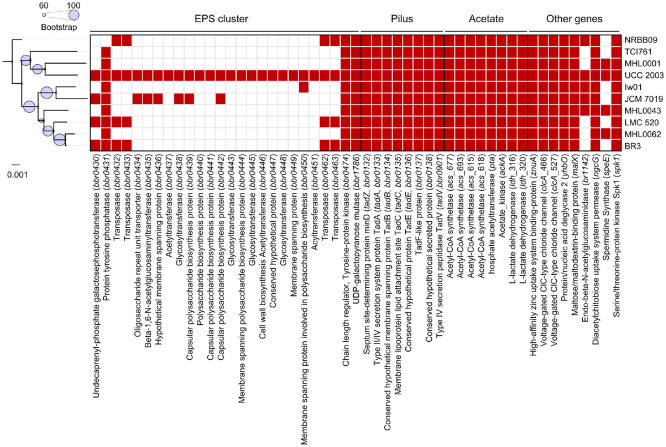
Comparison of immune-related genes among strains within the subclade. The left panel shows reconstructed SNP tree of subclade genomes. Bootstrap values were calculated with 100 replicates and are shown as light purple circles. Scale bar indicates substitutions per site. The binary matrix showing the presence (red) or absence (white) of immune-related genes. Rows represent strains, and columns denote functional gene categories (top) and corresponding gene names/products (bottom).

To compare immunologically relevant genes in the genomes of nine strains, an SNP tree was reconstructed using *B. breve* NRBB09 as the outgroup. The concatenated sequences of 51 selected genes were aligned, and the presence or absence of these genes across genomes was analyzed (Fig. 4). Strain MHL0001 clustered with UCC 2003, while MHL0043 and MHL0062 clustered with BR3 and LMC 520. All genes within the EPS biosynthesis cluster were exclusively present in UCC 2003, though some genes were detected in other strains. Genes involved in pilus formation and acetate production were detected in all strains, along with genes such as *znuA*, *clcA*, *ynbO*, *malX*, *ngcG*, and *spk1*. The speE gene, associated with anti-inflammatory macrophage activity, was detected in both our isolated strains and UCC 2003.

**Fig. 5:**
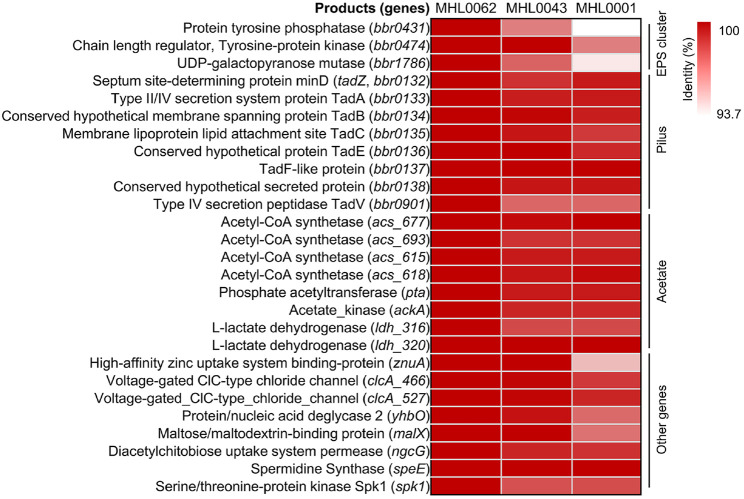
Heatmap of gene identity for shared immune-related genes among isolates. Pairwise identities of 27 shared immune-related genes were calculated using MHL0062 as the reference. Heatmap colors represent percentage identity, ranging from 93.7% to 100% identity.

Although MHL0001 clustered distinctly from MHL0043 and MHL0062, the immune-related genes profiles were highly similar among the isolated strains. To further analyze, the sequence identities of immune-related genes were compared across all three isolates (Fig. 5). A total of 27 genes were shared among the isolates, with genes in MHL0062 used as the reference for sequence identity calculations. The sequence identities of genes between MHL0001 and MHL0062 were more divergent compared to those between MHL0043 and MHL0062. This finding aligned with the clustering pattern in the SNP tree. Low sequence homology between MHL0001 and MHL0062 was detected in genes associated with the EPS cluster, *znuA*, *yhbO*, *malX*, and *spk1*.

### Differential Anti-Inflammatory Effects of three *B. breve* Isolates

**Fig. 6:**
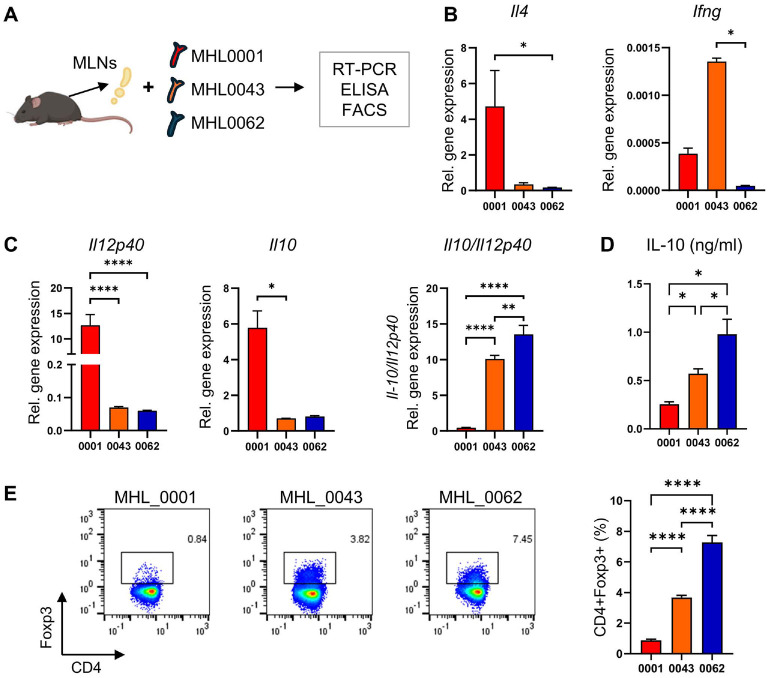
Host immune response to isolated *B. breve* strains. (**A**) Experimental design schematic. MLN cells from C57BL/6 mice were co-cultured with *B. breve* strains, and immune responses were analyzed by RT-PCR, ELISA, and flow cytometry. (**B**) IL-4 and IFN-γ expression in MLN cells after 24 h of co-culture. Relative expression was normalized to β-actin and to control cells without bacteria. (**C**) *Il12p40* and *Il10* expressions and their ratio in MNL cells exposed to isolates. (**D**) IL-10 secretion in culture supernatant quantified by ELISA after 48 h of co-culture. (**E**) Frequencies of CD4+Foxp3+ T_reg_ cells detected by flow cytometry after 24 h of co-culture. Representative FACS plot and quantification from three independent replicates pre strain are shown. Data are presented as mean ± SD from two independent experiments. Statistical significance was determined by one-way ANOVA test. ****p < 0.0001, ***p < 0.001, **p < 0.01, *p < 0.05.

The pathogenesis of AD is primarily driven by excessive Type II immunity, accompanied by impaired Type I responses. Representative cytokines for these pathways, IL-4 or IFN-γ, can serve as indicators of immune imbalance. To examine host immune responses to *B. breve* strains, isolates were co-cultured with MLN-derived cells (Fig. 6A). Immune cells exposed to MHL0001 showed significantly higher *Il4* expression compared with those treated with MHL0043 or MHL0062. In contrast, *Ifng* expression was highest in cells treated with MHL0043, which was isolated from a remission state, whereas cells exposed to MHL0062 displayed lower *Ifng* expression than those treated with MHL0001 (Fig. 6B). These Type I and Type II cytokine profiles closely reflected the pathological status of AD in infants.

Anti-inflammatory responses, mediated by IL-10 and CD4^+^ regulatory T (T_reg_) cells expressing *Foxp3*, are well-recognized mechanisms for suppressing dysregulated immunity. To determine the anti-inflammatory potential of the isolates, IL-10 expression and T_reg_ frequencies were measured in MLN cells following co-culture with each *B. breve* strain. In response to bacterial simulation, host cells produced both inflammatory and anti-inflammatory cytokines, and the ratio of these cytokines reflected the overall immune response. IL-12p40 was used as the representative pro-inflammatory cytokine [50]. Notably, the *Il10/Il12p40* expression ratio and IL-10 production were higher in MLN cells treated with MHL0062 compared with those treated with MHL0043 or MHL0001 (Fig. 6C and 6D). Consistently, T_reg_ frequencies were also increased in MHL0062-treated cells relative to MHL0043- or MHL0001-treated cells (Fig. 6E).

## Discussion

This study compared the genomes and immune-modulatory functions of *B. breve* strains isolated from infants with and without AD, alongside publicly available complete genomes. Although overall genome similarity was high, isolates differed in the sequence homology of several immune-related genes, and these genomic differences were reflected in distinct immune response *in vitro*. These findings suggest that *B. breve* strain in AD and non-AD infants may play functionally divergent roles.

In our previous studies, nine *Bifidobacterium* species were identified in the infant gut microbiome [7, 9]. Among these, the relative abundances of *B. breve* and *B. longum* differed significantly between AD and non-AD groups (*p* < 0.05), while no significant differences were observed for the other *Bifidobacterium* species. Notably, the relative abundance of *B. breve* was higher in the AD group, highlighting the need for detailed evaluation of the beneficial effects of *B. breve* that have been reported in reported in previous studies [22, 24]. Strain-level analysis using StrainPhlAn revealed compositional difference in *B. breve* between AD and non-AD groups, including AD-specific strains. These results underscore the importance of strain level resolution in microbiome studies [52, 53].

Although the three isolates displayed high genomic similarity, SNP-based phylogeny showed that MHL0062 clustered with the BR3, a strain know for anti-inflammatory effects [54], whereas MHL0001 and MHL0043 formed a separate group. Five additional strains were grouped with the isolates in this study. While beneficial and therapeutic effects have been reported for lw01 and BR3 strains [54, 55], experimental evidence of the other strains (TCI761, JCM 7019, and LMC 520) remains limited. Comparative analysis of isolates and reference strains within the same subcluster, including the well-characterized UCC 2003 strain [37], revealed no ARGs, but did identify differences in the number of CRISPR, mobile elements, HGT events, and prophage content. These findings suggest that the strains have undergone distinct evolutionary trajectories independent of ARGs.

Variation within the EPS gene cluster was notable, with the complete cluster present only in UCC 2003. EPSs, secreted extracellular polysaccharides, play a vital role in host-microbe interactions [56] and are considered key contributors to the probiotic mechanisms of *Bifidobacterium* [39]. While partial EPS cluster genes were identified in other strains, variations in EPS-related genes have been reported previously [57]. Further experiments are therefore needed to confirm EPSs production in the isolates.

Genes related to pilus formation and acetate production were detected across all nine strains. Pilus formation facilitates colonization in the gut environment by mediating adherence to the host tissues, potentially enhancing anti-inflammatory effects [37]. Acetate, a short-chain fatty acid (SCFA), is well established as a regulator of host immune responses and intestinal inflammation [58, 59]. In contrast, genes encoding endo-beta-N-aceytylglucosaminidase and *speE* varied among the strains, whereas other immune-related genes were consistently detected. These observations indicate that although isolates harbor several genetic determinants linked to beneficial effects, genomic content alone does not clearly differentiate AD- from non-AD-derived strains.

To bridge genomic variation and functional output, we examined sequence divergence in common immune-related genes. EPS cluster genes and *znuA*, *yhbO*, *malX*, and *spk1*, showed the greatest divergence between MHL0062 (from a non-AD infant) and MHL0001 (from an AD infant). The *znuA* gene, essential for zinc uptake, contributes to probiotic absorption, immune function, intestinal barrier integrity, and electrolyte transport [60, 61]. The *yhbO* gene is implicated in oxidative stress reduction through repair of damaged proteins and nucleic acids, thereby alleviating inflammation [62, 63]. The *malX* gene, involved in carbohydrate metabolism, may support a balanced gut microbiota [46]. The *spk1* gene, encoding a serine/threonine protein kinase, regulates signaling pathways such as Wnt/β-catenin and NF-κB, which are central to inflammation and metabolic regulation [48, 64].

Gene sequence similarity was higher between MHL0062 and MHL0043 (from an infant in remission) than between MHL0062 and MHL0001. This suggests that sequence difference in immune-related genes between MHL0062 and MHL0001 may underlie strain-specific roles of *B. breve* and contribute to its increased abundance in AD infants. Immune assays confirmed that host cells exhibited greater anti-inflammatory responses to MHL0062 than to MHL0001, as evidenced by increased IL-10 production and higher T_reg_ frequencies. Furthermore, gene expression of *Ifng*, the *Il10/Il12p40* ratio, IL-10 production, and T_reg_ percentages were all upregulated in MLN cells treated with MHL0043 compared to MHL0001. Notably, MHL0043, isolated from an infant in AD remission, induced both enhanced Type I immune response and anti-inflammatory effects, suggesting that strain-specific immune modulation may reflect disease remission status and recovery of immune balance. However, the mechanism by which *B. breve* strains influence gut immunity and shape microbial ecosystem requires further investigation.

In conclusion, our results show that the beneficial effects of *B. breve* are highly strain specific and shaped by subtle but functionally meaningful genomic variation. The presence of *B. breve*—or of genes associated with anti-inflammatory pathways—does not necessarily predict immune-modulatory capacity. Comprehensive analyses integrating genomics, gene expression, and host interaction studies will be critical for defining strain-level functions. These findings also provide a possible explanation for the increased abundance of *B. breve* in AD infants and caution against interpreting *Bifidobacterium* abundance as inherently beneficial without strain-level resolution.

## Supplemental Materials

Supplementary data for this paper are available on-line only at http://jmb.or.kr.



## Figures and Tables

**Table 1:** General features of six complete genomes and three isolated genomes of *Bifidobacterium breve*.

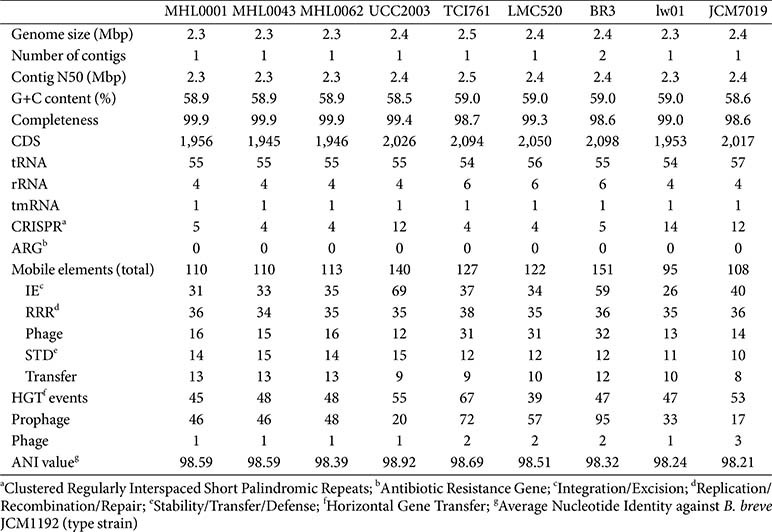

## References

[1] Asher MI, Montefort S, Bjorksten B, Lai CK, Strachan DP, Weiland SK (2006). Worldwide time trends in the prevalence of symptoms of asthma, allergic rhinoconjunctivitis, and eczema in childhood: ISAAC phases one and three repeat multicountry crosssectional surveys. Lancet.

[2] Facheris P, Jeffery J, Del Duca E, Guttman-Yassky E (2023). The translational revolution in atopic dermatitis: the paradigm shift from pathogenesis to treatment. Cell Mol. Immunol..

[3] Zhou H, Sun L, Zhang S, Zhao X, Gang X, Wang G (2021). The crucial role of early-life gut microbiota in the development of type 1 diabetes. Acta Diabetol..

[4] Stewart CJ, Ajami NJ, O'Brien JL, Hutchinson DS, Smith DP, Wong MC (2018). Temporal development of the gut microbiome in early childhood from the TEDDY study. Nature.

[5] Geuking MB, Cahenzli J, Lawson MA, Ng DC, Slack E, Hapfelmeier S, McCoy KD, Macpherson AJ (2011). Intestinal bacterial colonization induces mutualistic regulatory T cell responses. Immunity.

[6] Fujimura KE, Sitarik AR, Havstad S, Lin DL, Levan S, Fadrosh D (2016). Neonatal gut microbiota associates with childhood multisensitized atopy and T cell differentiation. Nat. Med..

[7] Lee MJ, Kang MJ, Lee SY, Lee E, Kim K, Won S (2018). Perturbations of gut microbiome genes in infants with atopic dermatitis according to feeding type. J. Allergy Clin. Immunol..

[8] Paller AS, Kong HH, Seed P, Naik S, Scharschmidt TC, Gallo RL, Luger T, Irvine AD (2019). The microbiome in patients with atopic dermatitis. J. Allergy Clin. Immunol..

[9] Lee MJ, Park YM, Kim B, Tae IH, Kim NE, Pranata M (2022). Disordered development of gut microbiome interferes with the establishment of the gut ecosystem during early childhood with atopic dermatitis. Gut Microbes.

[10] Roger LC, Costabile A, Holland DT, Hoyles L, McCartney AL (2010). Examination of faecal *Bifidobacterium* populations in breastand formula-fed infants during the first 18 months of life. Microbiology (Reading).

[11] Favier CF, Vaughan EE, De Vos WM, Akkermans AD (2002). Molecular monitoring of succession of bacterial communities in human neonates. Appl. Environ. Microbiol..

[12] Kalliomaki M, Kirjavainen P, Eerola E, Kero P, Salminen S, Isolauri E (2001). Distinct patterns of neonatal gut microflora in infants in whom atopy was and was not developing. J. Allergy Clin. Immunol..

[13] Abrahamsson TR, Jakobsson HE, Andersson AF, Bjorksten B, Engstrand L, Jenmalm MC. 2012. Low diversity of the gut microbiota in infants with atopic eczema. *J. Allergy Clin. Immunol.* **129:** 434-40, 440 e1-2. 10.1016/j.jaci.2011.10.025 22153774

[14] Ismail IH, Oppedisano F, Joseph SJ, Boyle RJ, Licciardi PV, Robins-Browne RM, Tang ML (2012). Reduced gut microbial diversity in early life is associated with later development of eczema but not atopy in high-risk infants. Pediatr. Allergy Immunol..

[15] Zheng H, Liang H, Wang Y, Miao M, Shi T, Yang F (2016). Altered gut microbiota composition associated with eczema in infants. PLoS One.

[16] Zimmermann P, Messina N, Mohn WW, Finlay BB, Curtis N (2019). Association between the intestinal microbiota and allergic sensitization, eczema, and asthma: a systematic review. J. Allergy Clin. Immunol..

[17] Galazzo G, van Best N, Bervoets L, Dapaah IO, Savelkoul PH, Hornef MW (2020). Development of the microbiota and associations with birth mode, diet, and atopic disorders in a longitudinal analysis of stool samples, collected from infancy through early childhood. Gastroenterology.

[18] Depner M, Taft DH, Kirjavainen PV, Kalanetra KM, Karvonen AM, Peschel S (2020). Maturation of the gut microbiome during the first year of life contributes to the protective farm effect on childhood asthma. Nat. Med..

[19] Ta LDH, Chan JCY, Yap GC, Purbojati RW, Drautz-Moses DI, Koh YM (2020). A compromised developmental trajectory of the infant gut microbiome and metabolome in atopic eczema. Gut Microbes.

[20] Ismail IH, Boyle RJ, Licciardi PV, Oppedisano F, Lahtinen S, Robins-Browne RM (2016). Early gut colonization by *Bifidobacterium breve* and *B. catenulatum* differentially modulates eczema risk in children at high risk of developing allergic disease. Pediatr. Allergy Immunol..

[21] Natividad JM, Hayes CL, Motta JP, Jury J, Galipeau HJ, Philip V (2013). Differential induction of antimicrobial REGIII by the intestinal microbiota and *Bifidobacterium breve* NCC2950. Appl. Environ. Microbiol..

[22] van de Pol MA, Lutter R, Smids BS, Weersink EJ, van der Zee JS (2011). Synbiotics reduce allergen-induced T-helper 2 response and improve peak expiratory flow in allergic asthmatics. Allergy.

[23] Motei DE, Beteri B, Hepsomali P, Tzortzis G, Vulevic J, Costabile A (2023). Supplementation with postbiotic from *Bifidobacterium breve* BB091109 improves inflammatory status and endocrine function in healthy females: a randomized, double-blind, placebocontrolled, parallel-groups study. Front. Microbiol..

[24] Sagar S, Morgan ME, Chen S, Vos AP, Garssen J, van Bergenhenegouwen J (2014). *Bifidobacterium breve* and *Lactobacillus rhamnosus* treatment is as effective as budesonide at reducing inflammation in a murine model for chronic asthma. Respir. Res..

[25] Blanco-Miguez A, Beghini F, Cumbo F, McIver LJ, Thompson KN, Zolfo M (2023). Extending and improving metagenomic taxonomic profiling with uncharacterized species using MetaPhlAn 4. Nat. Biotechnol..

[26] Truong DT, Tett A, Pasolli E, Huttenhower C, Segata N (2017). Microbial strain-level population structure and genetic diversity from metagenomes. Genome Res..

[27] Chalita M, Kim YO, Park S, Oh HS, Cho JH, Moon J (2024). EzBioCloud: a genome-driven database and platform for microbiome identification and discovery. Int. J. Syst. Evol. Microbiol..

[28] Seemann T (2014). Prokka: rapid prokaryotic genome annotation. Bioinformatics.

[29] Couvin D, Bernheim A, Toffano-Nioche C, Touchon M, Michalik J, Neron B (2018). CRISPRCasFinder, an update of CRISRFinder, includes a portable version, enhanced performance and integrates search for Cas proteins. Nucleic Acids Res..

[30] Vernikos GS, Parkhill J (2006). Interpolated variable order motifs for identification of horizontally acquired DNA: revisiting the *Salmonella* pathogenicity islands. Bioinformatics.

[31] Brown CL, Mullet J, Hindi F, Stoll JE, Gupta S, Choi M (2022). mobileOG-db: a manually curated database of protein families mediating the life cycle of bacterial mobile genetic elements. Appl. Environ. Microbiol..

[32] Starikova EV, Tikhonova PO, Prianichnikov NA, Rands CM, Zdobnov EM, Ilina EN, Govorun VM (2020). Phigaro: high-throughput prophage sequence annotation. Bioinformatics.

[33] Guo J, Bolduc B, Zayed AA, Varsani A, Dominguez-Huerta G, Delmont TO (2021). VirSorter2: a multi-classifier, expert-guided approach to detect diverse DNA and RNA viruses. Microbiome.

[34] Alcock BP, Raphenya AR, Lau TTY, Tsang KK, Bouchard M, Edalatmand A (2020). CARD 2020: antibiotic resistome surveillance with the comprehensive antibiotic resistance database. Nucleic Acids Res..

[35] Lee I, Ouk Kim Y, Park SC, Chun J (2016). OrthoANI: an improved algorithm and software for calculating average nucleotide identity. Int. J. Syst. Evol. Microbiol..

[36] Shakya M, Ahmed SA, Davenport KW, Flynn MC, Lo CC, Chain PSG (2020). Standardized phylogenetic and molecular evolutionary analysis applied to species across the microbial tree of life. Sci. Rep..

[37] O'Connell Motherway M, Zomer A, Leahy SC, Reunanen J, Bottacini F, Claesson MJ (2011). Functional genome analysis of *Bifidobacterium breve* UCC2003 reveals type IVb tight adherence (Tad) pili as an essential and conserved host-colonization factor. Proc. Natl. Acad. Sci. USA.

[38] Ruiz L, Bottacini F, Boinett CJ, Cain AK, O'Connell-Motherway M, Lawley TD, van Sinderen D (2017). The essential genomic landscape of the commensal *Bifidobacterium breve* UCC2003. Sci. Rep..

[39] Fanning S, Hall LJ, van Sinderen D (2012). *Bifidobacterium breve* UCC2003 surface exopolysaccharide production is a beneficial trait mediating commensal-host interaction through immune modulation and pathogen protection. Gut Microbes.

[40] Nandakumar KS, Collin M, Happonen KE, Lundstrom SL, Croxford AM, Xu B (2018). Streptococcal endo-beta-Nacetylglucosaminidase suppresses antibody-mediated inflammation in vivo. Front. Immunol..

[41] Alessandri G, Milani C, Duranti S, Mancabelli L, Ranjanoro T, Modica S (2019). Ability of bifidobacteria to metabolize chitinglucan and its impact on the gut microbiota. Sci. Rep..

[42] He J, Zhang P, Shen L, Niu L, Tan Y, Chen L (2020). Short-chain fatty acids and their association with signalling pathways in inflammation, glucose and lipid metabolism. Int. J. Mol. Sci..

[43] Jarosz M, Olbert M, Wyszogrodzka G, Mlyniec K, Librowski T (2017). Antioxidant and anti-inflammatory effects of zinc. Zincdependent NF-kappaB signaling. Inflammopharmacology.

[44] Xiang NL, Liu J, Liao YJ, Huang YW, Wu Z, Bai ZQ (2016). Abrogating ClC-3 inhibits LPS-induced inflammation via blocking the TLR4/NF-kappaB pathway. Sci. Rep..

[45] Abdallah J, Mihoub M, Gautier V, Richarme G (2016). The DJ-1 superfamily members YhbO and YajL from *Escherichia coli* repair proteins from glycation by methylglyoxal and glyoxal. Biochem. Biophys. Res. Commun..

[46] Almutairi R, Basson AR, Wearsh P, Cominelli F, Rodriguez-Palacios A (2022). Validity of food additive maltodextrin as placebo and effects on human gut physiology: systematic review of placebo-controlled clinical trials. Eur. J. Nutr..

[47] Niechcial A, Schwarzfischer M, Wawrzyniak M, Atrott K, Laimbacher A, Morsy Y (2023). Spermidine ameliorates colitis via induction of anti-inflammatory macrophages and prevention of intestinal dysbiosis. J. Crohns Colitis.

[48] Cortes-Vieyra R, Silva-Garcia O, Gomez-Garcia A, Gutierrez-Castellanos S, Alvarez-Aguilar C, Baizabal-Aguirre VM (2021). Glycogen synthase kinase 3beta modulates the inflammatory response activated by bacteria, viruses, and parasites. Front. Immunol..

[49] Edgar RC (2010). Search and clustering orders of magnitude faster than BLAST. Bioinformatics.

[50] Kim JE, Sharma A, Sharma G, Lee SY, Shin HS, Rudra D, Im SH (2019). *Lactobacillus pentosus* modulates immune response by inducing IL-10 producing Tr1 cells. Immune Netw..

[51] Bottacini F, Morrissey R, Esteban-Torres M, James K, van Breen J, Dikareva E (2018). Comparative genomics and genotypephenotype associations in *Bifidobacterium breve*. Sci. Rep..

[52] Valles-Colomer M, Blanco-Miguez A, Manghi P, Asnicar F, Dubois L, Golzato D (2023). The person-to-person transmission landscape of the gut and oral microbiomes. Nature.

[53] Schmidt TSB, Raes J, Bork P (2018). The human gut microbiome: from association to modulation. Cell.

[54] Park IS, Kim JH, Yu J, Shin Y, Kim K, Kim TI, Kim SW, Cheon JH (2023). *Bifidobacterium breve* CBT BR3 is effective at relieving intestinal inflammation by augmenting goblet cell regeneration. J. Gastroenterol. Hepatol..

[55] Li Q, Li Y, Wang Y, Xu L, Guo Y, Wang Y (2021). Oral administration of *Bifidobacterium breve* promotes antitumor efficacy via dendritic cells-derived interleukin 12. Oncoimmunology.

[56] Laino J, Villena J, Kanmani P, Kitazawa H (2016). Immunoregulatory effects triggered by lactic acid bacteria exopolysaccharides: new insights into molecular interactions with host cells. Microorganisms.

[57] Ferrario C, Milani C, Mancabelli L, Lugli GA, Duranti S, Mangifesta M (2016). Modulation of the eps-ome transcription of bifidobacteria through simulation of human intestinal environment. FEMS Microbiol. Ecol..

[58] Aoki R, Kamikado K, Suda W, Takii H, Mikami Y, Suganuma N (2017). A proliferative probiotic *Bifidobacterium* strain in the gut ameliorates progression of metabolic disorders via microbiota modulation and acetate elevation. Sci. Rep..

[59] Tedelind S, Westberg F, Kjerrulf M, Vidal A (2007). Anti-inflammatory properties of the short-chain fatty acids acetate and propionate: a study with relevance to inflammatory bowel disease. World J. Gastroenterol..

[60] Ahmadipour S, Mohsenzadeh A, Alimadadi H, Salehnia M, Fallahi A (2019). Treating viral diarrhea in children by probiotic and zinc supplements. Pediatr. Gastroenterol. Hepatol. Nutr..

[61] Shah M, Zaneb H, Masood S, Khan RU, Ashraf S, Sikandar A (2019). Effect of dietary supplementation of zinc and multi-microbe probiotic on growth traits and alteration of intestinal architecture in broiler. Probiotics Antimicrob. Proteins.

[62] Iyer H, Talbot WS (2024). The Cl- transporter ClC-7 is essential for phagocytic clearance by microglia. J. Cell Sci..

[63] Sun Y, Wang X, Li L, Zhong C, Zhang Y, Yang X, Li M, Yang C (2024). The role of gut microbiota in intestinal disease: from an oxidative stress perspective. Front. Microbiol..

[64] Ghadimi D, Nielsen A, Hassan MFY, Fölster-Holst R, de Vrese M, Heller KJ (2019). Modulation of GSK - 3β/β - catenin cascade by commensal bifidobateria plays an important role for the inhibition of metaflammation-related biomarkers in response to LPS or non-physiological concentrations of fructose: an *in vitro* study. PharmaNutrition.

