# Supplementary material for: Strain-Level Differences of Bifidobacterium breve in the Gut Microbiome between Infants with and without Atopic Dermatitis: Insights from Genome Analysis and Immune Assays
Source: J Microbiol Biotechnol. 2025 Nov 26;35:e2509032. doi: 10.4014/jmb.2509.09032 (PMC12685581; doi:10.4014/jmb.2509.09032)
Supplement: Supplementary file 1 [file jmb-35-e2509032-supple.pdf]

J. Microbiol. Biotechnol. 2025. 35: e2509032

https://doi.org/10.4014/jmb.2509.09032

Supplementary Tables

Strain-level differences of *Bifidobacterium breve* in the gut microbiome between infants with and without atopic dermatitis: Insights from genome analysis and immune assays

Imchang Lee[1], Seong Hee Kim[2], Min-Jung Lee[3], Ara Oh[4], Yun Kyung Lee[4], Kwang Jun Lee[5*], Bong-Soo Kim[3*]

[1]Division of Infectious Diseases, Department of Internal Medicine, Hallym University Chuncheon Sacred Heart Hospital, Hallym Univeristy College of Medicine, Chuncheon, Gangwon-do, Republic of Korea

[2]Department of Life Science, Hallym University, Chuncheon, Gangwon-do, Republic of Korea

[3]Department of Nutritional Science and Food Management, Ewha Womans University, Seoul, Republic of Korea

[4]Department of Integrated Biomedical Science, Soonchunhyang Institute of Medi-Bioscience, Soonchunhyang University, Cheonan, Republic of Korea

[5]Division of Zoonotic and Vector Brone Disease Research, National Institute of Health, Korea Disease Control and Prevention Agency, Cheongju, Chungbuk-do, Republic of Korea

Table S1. Genome features of 49 *Bifidobacterium breve* complete genomes were used in this study. Sequences and information of each strain were obtained from NCBI database.

Strain	Size (bp)	GC content (%)	Gene counts (n)	Source	Host	Country	Bioproject number
ACS-071-V-Sch8b	2,327,492	58.5	1,998	Vagina	Human	USA	PRJNA51077
UCC2003	2,422,684	58.5	2,082	Fecal	Infant	Ireland	PRJNA13487
12L	2,244,624	59.0	1,923	Human milk	Human	Italy	PRJNA214949
JCM 7017	2,288,919	58.5	1,964	Fecal	Infant	Japan	PRJNA214950
JCM 7019	2,359,009	58.5	2,091	Fecal	Adult	Japan	PRJNA214951
NCFB 2258	2,315,904	58.5	1,994	Fecal	Infant	UK	PRJNA214954
689b	2,331,707	58.5	2,013	Fecal	Infant	Italy	PRJNA214955
S27	2,294,458	58.5	1,966	Fecal	Infant	Germany	PRJNA214956
JCM 1192	2,269,415	59.0	2,004	Fecal	Infant	Japan	PRJDB57
BR3	2,426,006	59.0	2,177	Fecal	Infant	South Korea	PRJNA270903
LMC520	2,403,402	59.0	2,122	Environment	_	South Korea	PRJNA369572
UCC2003	2,422,684	58.5	2,082	Fecal	Infant	Ireland	PRJNA13487
DRBB26	2,396,387	58.5	2,081	Fecal	Infant	Netherlands	PRJNA384072
NRBB01	2,269,404	59.0	2,010	Fecal	Infant	Netherlands	PRJNA384038
NRBB02	2,289,884	58.5	1,975	Fecal	Infant	Netherlands	PRJNA384042
NRBB04	2,324,647	58.5	2,006	Fecal	Infant	Netherlands	PRJNA384043
NRBB11	2,377,562	58.5	2,023	Fecal	Infant	Netherlands	PRJNA384046
NRBB09	2,265,557	58.5	1,967	Fecal	Infant	Netherlands	PRJNA384044
NRBB57	2,510,381	59.5	2,252	Fecal	Infant	Netherlands	PRJNA384069
NRBB50	2,409,058	59.0	2,135	Fecal	Infant	Netherlands	PRJNA384047
NRBB52	2,379,672	59.0	2,092	Fecal	Infant	Netherlands	PRJNA384049
NRBB51	2,402,272	59.0	2,064	Fecal	Infant	Netherlands	PRJNA384048
NRBB56	2,425,122	59.0	2,095	Fecal	Infant	Netherlands	PRJNA384068
DRBB27	2,435,083	59.0	2,195	Fecal	Infant	Netherlands	PRJNA384073

017W439	2,301,422	58.5	2,019	Fecal	Infant	Ireland	PRJNA384113
215W447a	2,589,602	59.5	2,349	Fecal	Infant	Ireland	PRJNA384129
DRBB28	2,462,170	59.0	2,204	Fecal	Infant	Netherlands	PRJNA384074
180W83	2,273,173	59.0	1,995	Fecal	Infant	Ireland	PRJNA384128
082W48	2,286,339	59.0	1,994	Fecal	Infant	Ireland	PRJNA384114
139W423	2,411,276	58.5	2,128	Fecal	Infant	Ireland	PRJNA384124
CNCM I-4321	2,464,852	59.0	2,216	Fecal	Infant	Netherlands	PRJNA384070
NRBB18	2,289,686	58.5	1,985	Fecal	Infant	Netherlands	PRJNA387666
NRBB19	2,289,726	58.5	1,985	Fecal	Infant	Netherlands	PRJNA387699
NRBB20	2,289,892	58.5	1,984	Fecal	Infant	Netherlands	PRJNA387670
NRBB27	2,289,838	58.5	1,986	Fecal	Infant	Netherlands	PRJNA387692
NRBB49	2,289,791	58.5	1,984	Fecal	Infant	Netherlands	PRJNA387696
DRBB29	2,435,086	59.0	2,196	Fecal	Infant	Netherlands	PRJNA387697
DRBB30	2,471,118	59.0	2,215	Fecal	Infant	Netherlands	PRJNA387698
NRBB08	2,289,759	58.5	1,984	Fecal	Infant	Netherlands	PRJNA387665
FDAARGOS_561	2,275,646	59.0	2,008	Clinical isolate	Human	_	PRJNA231221
lw01	2,313,172	59.0	2,026	Fecal	Infant	China	PRJNA507053
JSRL01	2,274,146	58.5	1,943	Fecal	Infant	South Korea	PRJNA579858
JR01	2,304,912	59.0	2,024	Fecal	Human	Sweden	PRJNA489428
JTL	2,289,549	58.5	1,956	Fecal	Infant	South Korea	PRJNA635210
BIF195	2,336,700	58.5	2,023	_	Human	_	PRJNA860779
1101A	2,371,121	59.0	2,065	Fecal	Children	Brazil	PRJNA633614
VSI11	2,536,081	59.0	2,357	Vagina	Human	USA	PRJNA934404
TCI761	2,460,420	59.0	2,161	_	Human	Taiwan	PRJNA1077123
NCTC11815	2,275,664	59.0	2,007	Fecal	Infant	UK	PRJEB6403

Table S2. Information of 51 genes with known immune-related functions in comparison.

Gene	Products	Class	Category	Reference Strain	Accession
bbr_0430	Undecaprenyl-phosphate galactosephosphotransferase	UDP-Galactosephosphotransferase	Eps	UCC2003	CP000303.1
bbr_0431	Protein tyrosine phosphatase	Protein tyrosine phosphatase	Eps	UCC2003	CP000303.1
bbr_0432	Transposase	Transposase	Eps	UCC2003	CP000303.1
bbr_0433	Transposase	Transposase	Eps	UCC2003	CP000303.1
bbr_0434	Oligosaccharide repeat unit transporter	Flippase	Eps	UCC2003	CP000303.1
bbr_0435	Beta-1,6-N-acetylglucosaminyltransferase	Glycosyltransferase	Eps	UCC2003	CP000303.1
bbr_0436	Hypothetical membrane spanning protein	Polymerase	Eps	UCC2003	CP000303.1
bbr_0437	Acetyltransferase	Acetyltransferase	Eps	UCC2003	CP000303.1
bbr_0438	Glycosyltransferase	Glycosyltransferase	Eps	UCC2003	CP000303.1
bbr_0439	Capsular polysaccharide biosynthesis protein	Glycosyltransferase	Eps	UCC2003	CP000303.1
bbr_0440	Polysaccharide biosynthesis protein	Glycosyltransferase	Eps	UCC2003	CP000303.1
bbr_0441	Capsular polysaccharide biosynthesis protein	Glycosyltransferase	Eps	UCC2003	CP000303.1
bbr_0442	Capsular polysaccharide biosynthesis protein	Glycosyltransferase	Eps	UCC2003	CP000303.1
bbr_0443	Glycosyltransferase	Glycosyltransferase	Eps	UCC2003	CP000303.1
bbr_0444	Membrane spanning polysaccharide biosynthesis protein	Flippase	Eps	UCC2003	CP000303.1
bbr_0445	Glycosyltransferase	Glycosyltransferase	Eps	UCC2003	CP000303.1
bbr_0446	Acetyltransferase (cell wall biosynthesis)	Acetyltransferase	Eps	UCC2003	CP000303.1
bbr_0447	Conserved hypothetical protein	Conserved hypothetical protein	Eps	UCC2003	CP000303.1
bbr_0448	Glycosyltransferase	Glycosyltransferase	Eps	UCC2003	CP000303.1

bbr_0449	Hypothetical membrane spanning protein	Membrane spanning protein	Eps	UCC2003	CP000303.1
bbr_0450	Membrane spanning protein involved in polysaccharide biosynthesis protein	Polymerase	Eps	UCC2003	CP000303.1
bbr_0451	Acyltransferase	Acyltransferase	Eps	UCC2003	CP000303.1
bbr_0462	Transposase	Transposase	Eps	UCC2003	CP000303.1
bbr_0463	Transposase	Transposase	Eps	UCC2003	CP000303.1
bbr_0474	Capsular polysaccharide biosynthesis protein (Chain length determinant)	Kinase	Eps	UCC2003	CP000303.1
bbr_1786	Glycosyltransferase	Glycosyltransferase	Eps	UCC2003	CP000303.1
bbr_0132	Septum site-determining protein minD	ATPase	Pilus	UCC2003	CP000303.1
bbr_0133	Type II/IV secretion system protein TadA	NTPase	Pilus	UCC2003	CP000303.1
bbr_0134	Conserved hypothetical membrane spanning protein TadB	Membrane protein	Pilus	UCC2003	CP000303.1
bbr_0135	Membrane lipoprotein lipid attachment site TadC	Lipoprotein	Pilus	UCC2003	CP000303.1
bbr_0136	Conserved hypothetical protein TadE	Conserved hypothetical protein	Pilus	UCC2003	CP000303.1
bbr_0137	TadF-like protein	Hypothetical protein	Pilus	UCC2003	CP000303.1
bbr_0138	Conserved hypothetical secreted protein	Conserved hypothetical protein	Pilus	UCC2003	CP000303.1
bbr_0901	Type IV secretion peptidase TadV	Peptidase	Pilus	UCC2003	CP000303.1
acs_677	Acetyl-CoA synthetase	Synthetase	Acetate	DSM 20213	ACCG02000009.1
acs_693	Acetyl-CoA synthetase	Synthetase	Acetate	DSM 20213	ACCG02000009.1
acs_615	Acetyl-CoA synthetase	Synthetase	Acetate	DSM 20213	ACCG02000009.1
acs_618	Acetyl-CoA synthetase	Synthetase	Acetate	DSM 20213	ACCG02000009.1
pta	Phosphate acetyltransferase	Acetyltransferase	Acetate	DSM 20213	ACCG02000009.1
ackA	Acetate kinase	Kinase	Acetate	DSM 20213	ACCG02000009.1

ldh_316	L-lactate dehydrogenase	Dehydrogenase	Acetate	DSM 20213	ACCG02000009.1
ldh_320	L-lactate dehydrogenase	Dehydrogenase	Acetate	DSM 20213	ACCG02000009.1
znuA	High-affinity zinc uptake system binding-protein	Transporter	Other gene	DSM 20213	ACCG02000009.1
clcA_466	Voltage-gated ClC-type chloride channel ClcB, clcA - H(+)/Cl(-) exchange transporter_ClcA	Transporter	Other gene	DSM 20213	ACCG02000009.1
clcA_527	Voltage-gated ClC-type chloride channel ClcB, clcA - H(+)/Cl(-) exchange transporter_ClcA	Transporter	Other gene	DSM 20213	ACCG02000009.1
yhbO	Protein/nucleic acid deglycase 2	Deglycase	Other gene	DSM 20213	ACCG02000009.1
malX	Maltose/maltodextrin-binding protein	Transporter	Other gene	DSM 20213	ACCG02000009.1
bbr_1142	Endo-beta-N-acetylglucosaminidase	Acetylglucosaminidase	Other gene	UCC2003	CP000303.1
ngcG	Diacetylchitobiose uptake system permease	Permease	Other gene	UCC2003	CP000303.1
speE	Spermidine synthase	Transferase	Other gene	JR01	CP040931.1
spk1	Serine/threonine-protein kinase	Kinase	Other gene	JG_Bg463	CABHNR010000000

Table S3. Primer sequences for RT-PCR

Primer	Forward (5′ to 3′)	Reverse (5′ to 3′)
β-actin	TTCGTTGCCGGTCCACA	ACCAGCGCAGCGATATCG
IL-4	GGTCTCAACCCCCAGCTAGT	GCCGATGATCTCTCTCAAGTGAT
IFN-γ	CCTGCGGCCTAGCTCTGA	GCCATGAGGAAGAGCTGCA
IL12p40	TGGTTTGCCATCGTTTTGCTG	ACAGGTGAGGTTCACTGTTTCT
IL-10	CCTCAGGATGAGGCTGAG	GCTCCACTGCCTTGCTCTTATT

Table S4. Summary of annotated CDS unique to each strain and shared between two strains.

Genes	MHL0001	MHL0043	MHL0062	Product
yraA	+			Putative cysteine protease YraA
evgA	+			DNA-binding transcriptional activator EvgA
araE_2	+			Arabinose-proton symporter
araE_1	+			Arabinose-proton symporter
ams	+			Amylosucrase
ytrF	+			ABC transporter permease YtrF
cheB	+			Protein-glutamate methylesterase/protein-glutamine glutaminase
cytR_1	+			HTH-type transcriptional repressor CytR
cytR_2	+			HTH-type transcriptional repressor CytR
tagH	+			Teichoic acids export ATP-binding protein TagH
galS	+			HTH-type transcriptional regulator GalS
yknY_3	+			Putative ABC transporter ATP-binding protein YknY
fdxA	+			Ferredoxin
yfeA	+			Periplasmic chelated iron-binding protein YfeA
oppA_3	+			Oligopeptide-binding protein OppA
hypBA1	+			Non-reducing end beta-L-arabinofuranosidase
malX_2	+			PTS system maltose-specific EIICB component
malX_1	+			Maltose/maltodextrin-binding protein
clcA_2	+			H(+)/Cl(-) exchange transporter ClcA
clcA_1	+			H(+)/Cl(-) exchange transporter ClcA
rep_2	+			ATP-dependent DNA helicase Rep
rep_1	+			ATP-dependent DNA helicase Rep
lysA_1		+		Diaminopimelate decarboxylase
lysA_2		+		Diaminopimelate decarboxylase
priA		+		putative primosomal protein N'
oppF_3		+		Oligopeptide transport ATP-binding protein OppF
treC		+		Trehalose-6-phosphate hydrolase
menH_3		+		2-succinyl-6-hydroxy-2,4-cyclohexadiene-1-carboxylate synthase
ddl		+		D-alanine--D-alanine ligase
glgE		+		Alpha-1,4-glucan:maltose-1-phosphate maltosyltransferase
hisA		+		1-(5-phosphoribosyl)-5-[(5-phosphoribosylamino)methylideneamino] imidazole-4-

				carboxamide isomerase
mpa		+		Proteasome-associated ATPase
nifH		+		Nitrogenase iron protein
thrB_1			+	Homoserine kinase
thrB_2			+	Homoserine kinase
fabG_1			+	3-oxoacyl-[acyl-carrier-protein] reductase FabG
fabG_2			+	3-oxoacyl-[acyl-carrier-protein] reductase FabG
cycB_1			+	Cyclodextrin-binding protein
cycB_2			+	Cyclodextrin-binding protein
menD_1			+	2-succinyl-5-enolpyruvyl-6-hydroxy-3-cyclohexene-1-carboxylate synthase
menD_2			+	2-succinyl-5-enolpyruvyl-6-hydroxy-3-cyclohexene-1-carboxylate synthase
btuD_19			+	Vitamin B12 import ATP-binding protein BtuD
bioM			+	Biotin transport ATP-binding protein BioM
bglA			+	Beta-glucosidase A
bglB			+	Thermostable beta-glucosidase B
rbsA_1			+	Ribose import ATP-binding protein RbsA
rbsA_2			+	Ribose import ATP-binding protein RbsA
atpFH			+	ATP synthase subunit b-delta
degA_5			+	HTH-type transcriptional regulator DegA
ptsI_1			+	Phosphoenolpyruvate-protein phosphotransferase
ptsI_2			+	Phosphoenolpyruvate-protein phosphotransferase
pyrE			+	Orotate phosphoribosyltransferase
polA			+	DNA polymerase I
araE			+	Arabinose-proton symporter
ecfA3	+	+		Energy-coupling factor transporter ATP-binding protein EcfA3
xni	+	+		Flap endonuclease Xni
bglB_1	+	+		Beta-glucosidase B
bglB_2	+	+		Thermostable beta-glucosidase B
rbsA	+	+		Ribose import ATP-binding protein RbsA
pknA	+	+		Serine/threonine-protein kinase PknA
kstR2	+	+		HTH-type transcriptional repressor KstR2
atpH	+	+		ATP synthase subunit delta
nagK	+	+		N-acetyl-D-glucosamine kinase

ptsI	+	+		Phosphoenolpyruvate-protein phosphotransferase
cycB	+	+		Cyclodextrin-binding protein
malL_3	+	+		Oligo-1,6-glucosidase
ccpA_3	+	+		Catabolite control protein A
lacF_8	+	+		Lactose transport system permease protein LacF
bvgA	+	+		Virulence factors putative positive transcription regulator BvgA
rlmD	+	+		23S rRNA (uracil(1939)-C(5))-methyltransferase RlmD
ktrA	+	+		Ktr system potassium uptake protein A
menD	+	+		2-succinyl-5-enolpyruvyl-6-hydroxy-3-cyclohexene-1-carboxylate synthase
fabG	+	+		3-oxoacyl-[acyl-carrier-protein] reductase FabG
lolD	+	+		Lipoprotein-releasing system ATP-binding protein LolD
rsmE	+	+		Ribosomal RNA small subunit methyltransferase E
pyrE_1	+	+		Orotate phosphoribosyltransferase
pyrE_2	+	+		Orotate phosphoribosyltransferase
thrB	+	+		Homoserine kinase
ugpA	+	+		sn-glycerol-3-phosphate transport system permease protein UgpA
araG	+	+		Arabinose import ATP-binding protein AraG
lacG	+		+	Lactose transport system permease protein LacG
pknD_3	+		+	Serine/threonine-protein kinase PknD
acpS	+		+	Holo-[acyl-carrier-protein] synthase
ddlA	+		+	D-alanine--D-alanine ligase A
priA_2	+		+	putative primosomal protein N'
priA_1	+		+	Phosphoribosyl isomerase A
arc	+		+	Proteasome-associated ATPase
dkgA	+		+	2,5-diketo-D-gluconic acid reductase A
treS	+		+	Trehalose synthase/amylase TreS
smc_5	+		+	Chromosome partition protein Smc
lysA	+		+	Diaminopimelate decarboxylase
araQ_9	+		+	L-arabinose transport system permease protein AraQ
insK	+		+	Putative transposase InsK for insertion sequence element IS150
crcB_4	+		+	Putative fluoride ion transporter CrcB
glgE_1	+		+	Alpha-1,4-glucan:maltose-1-phosphate maltosyltransferase

glgE_2	+		+	Alpha-1,4-glucan:maltose-1-phosphate maltosyltransferase
oppD	+		+	Oligopeptide transport ATP-binding protein OppD
spk1		+	+	Serine/threonine-protein kinase PK-1
znuA		+	+	High-affinity zinc uptake system binding-protein ZnuA
purR_8		+	+	HTH-type transcriptional repressor PurR
rep		+	+	ATP-dependent DNA helicase Rep
cefD		+	+	Isopenicillin N epimerase
clcB		+	+	Voltage-gated ClC-type chloride channel ClcB
clcA		+	+	H(+)/Cl(-) exchange transporter ClcA
lnrK_3		+	+	Transcriptional regulatory protein LnrK
yhbO		+	+	Protein/nucleic acid deglycase 2
addA		+	+	ATP-dependent helicase/nuclease subunit A
galR		+	+	HTH-type transcriptional regulator GalR
btuD_18		+	+	Vitamin B12 import ATP-binding protein BtuD
btuD_17		+	+	Vitamin B12 import ATP-binding protein BtuD
cytR		+	+	HTH-type transcriptional repressor CytR
malX		+	+	Maltose/maltodextrin-binding protein
uvrA_4		+	+	UvrABC system protein A
frlR		+	+	putative fructoselysine utilization operon transcriptional repressor
ngcG		+	+	Diacetylchitobiose uptake system permease protein NgcG
hypBA1_2		+	+	Non-reducing end beta-L-arabinofuranosidase
hypBA1_1		+	+	Non-reducing end beta-L-arabinofuranosidase
